# Effects of social activity on health-related quality of life according to age and gender: an observational study

**DOI:** 10.1186/s12955-015-0331-4

**Published:** 2015-09-11

**Authors:** Hye Ki Park, Sung Youn Chun, Young Choi, Seo Yoon Lee, Seung Ju Kim, Eun-Cheol Park

**Affiliations:** Department of Public Health, Graduate School, Yonsei University, Seoul, Republic of Korea; Institute of Health Services Research, Yonsei University College of Medicine, Seoul, Republic of Korea; Department of Health Policy and Management, Graduate School of Public Health, Yonsei University, Seoul, Republic of Korea; Department of Preventive Medicine, Yonsei University College of Medicine, 50 Yonsei-ro, Seodaemun-gu, Seoul, 120-752 Republic of Korea

## Abstract

**Background:**

The aim of this study was to examine the relationship between types and amount of social activity and health-related quality of life according to gender and age group.

**Methods:**

This study used data from the Community Health Survey (CHS), which was collected in 2011 and consisted of 229,226 participants aged 19 or older. A linear mixed effects model was used to evaluate the factors influencing health-related quality of life among individuals tracked in the CHS and, in particular, to analyze the associations between the amount and types of social activities participated in and the EuroQol EQ-5D assessment.

**Results:**

We found that the average quality of life increased according to the amount of social activities individuals participated in (zero = 89.30, one = 93.28, two = 95.25, three = 96.27, four = 96.85). When people participated in one social activity, social activity was more strongly associated with EQ-5D in the elderly age group (males: 19–34 years = 0.195, 35–49 years = 0.642, 50–64 years = 1.716, ≥65 years = 4.408; females: 19–34 years = 0.170, 35–49 years = 0.502, 50–64 years = 1.411, ≥65 years = 4.180). More participation was positively associated with higher EQ-5D (one = 1.939, two = 2.377, three = 2.439, four = 2.515, p for trend < 0.0001). In females, those who participated in relationship organizations had a higher EQ-5D than those who participated in other types of social activities (Females ≥65 age group; Relationship = 4.373, Leisure = 2.620, Religion = 1.842, Charity = 1.544).

**Conclusion:**

There was a positive association between the increase in the number of social activities and increase in health-related quality of life, especially when evaluated in terms of type of social activities and health-related quality of life according to gender and age group.

## Introduction

Quality of life has now become a significantly important endpoint in medical care. The World Health Organization’s definition of health includes mental and social health along with physical health [1]. At the same time, there are studies that support the idea that participating in social activities increases the quality of life [2]. There are many types of social activities that are effective for enhancing the quality of life. A previous study found that religious social activities were positively associated with life satisfaction [3]. Close relationships with friends and family and participation in leisure activities are also positively associated with high quality of life [4]. In addition, participating in volunteer work is closely associated with quality of life [5]. Thus, there are many studies that have recognized the association between participating in social activities and better quality of life. However, to our knowledge, most of the previous studies focused on only one kind of social activity type [6]; thus, the differences in effectiveness between particular social activities were not investigated. Furthermore, previous studies focused on limited populations such as adolescents [7], the elderly [8], or physically or mentally disabled patients [9, 10]. Therefore, we undertook the present study to examine whether social activity was associated or not with health-related quality of life in the general population according to age and gender. Moreover, as we included four types of effective social activities (religion, relationship, leisure, and charity), we could determine the specific associations of each type of social activity on quality of life. Specifically, this study also aimed to evaluate the association between social activity and quality of life by gender and age group.

## Methods

### Data collection and participants

This study used data from Community Health Survey (CHS), which was collected in 2011. The target population of the CHS was individuals who were older than 18 years of age anywhere in the Republic of Korea. The total population included in that survey was 229,226 individuals; we omitted individuals who had missing data about educational status, perceived health status, perceived stress status, marital status, economic activity status, family income, depression diagnosis, chronic and acute disease or accident and addiction experience, hypertension diagnosis, diabetes mellitus diagnosis, cerebral infarction diagnosis, cardiac infarction diagnosis, angina pectoris diagnosis, arthritis diagnosis, osteoporosis diagnosis, tuberculosis diagnosis, asthma diagnosis, EuroQol EQ-VAS and EQ-5D. Therefore, we used 209,315 people (male 94,531, female 114,784) for this analysis.

The CHS included 239 questions on health behavior, physical characteristics, vaccination, morbidity, prescription use, accidents, addictions, activity level, quality of life, education, and economic status, etc. across 18 fields. The CHS had previously created a “sample extraction framework” used the entire country’s address data from the Ministry of Security and Public Administration and the housing types and the household data from the Ministry of Land, Infrastructure, and Transport. Subsequently, based on this framework, the CHS extracted about 900 participants from each community health center. After extracting the participants, a well- trained investigator visited each participant’s house to perform the survey. The investigators interviewed participants one-to-one and entered the data into a computer. The CHS has been approved and deliberated every year by the Institutional Review Board of Korea Centers for Disease Control and Prevention from 2010. The approval code for 2011 was 2011-05CON-04-C. All participants received an explanation about the CHS and completed a consent form before they participated in the research.

We stratified the population by age and gender in order to determine the most effective social activity specific to these age groups. We stratified individuals by age into the following groups: 19 to 34 years old, 35 to 49 years old, 50 to 64 years old, and older than 65 years. For educational status, we categorized into less high school, high school graduate and college graduate. Participants who dropped out of school at any point were placed in the lower educational status group. Perceived health status was categorized as either good or bad. Good condition included very good, good, or normal. Bad condition included bad or very bad. Perceived stress status was also categorized into two groups: yes (and the magnitude of the stress) or no. Marital status was categorized to three groups: 1) married, 2) divorced, separated, or widowed, and 3) single. Family income was divided into four levels. Co-morbidities included chronic and acute diseases or accidents and addictions, hypertension, diabetes mellitus, cerebral infarction, angina pectoris, cardiac infarction, tuberculosis, or asthma diagnosis. Co-morbidities were categorized as zero co-morbidity, one co-morbidity, two co-morbidities, and three or more co-morbidities.

### Health-related quality of life

For estimating health-related quality of life, we used the EQ-5D questionnaire which included questions about mobility; M, self-care; SC, usual activities; UA, pain/discomfort; PD and anxiety/depression; AD. Each of the indexes consisted of no problems; level 1, some/moderate problems; level 2, and severe problems; level 3 [11]. We analyzed five EQ-5D indexes calculated using a weight-scoring system according to the Centers for Disease Control and Prevention guidelines [12]: EQ-5D index = 1 - (0.05 + 0.096*M2 + 0.418*M3 + 0.046*SC2 + 0.136*SC3 + 0.051*UA2 + 0.208*UA3 + 0.037*PD2 + 0.151*PD3 + 0.043*AD2 + 0.158*AD3 + 0.05*N3). If the mobility level was 2 then “M2” was defined as 1; otherwise, “M2” was defined as 0. Similarly, if the self-care level was 3 then “SC3” was defined 1; otherwise, “SC3” was 0. If all EQ-5D indexes scored 1, then the weighted score became 1.

### Social activity

The CHS included four kinds of social activities: religion, relationship, leisure, and charity. If an individual regularly participated in a social activity at least once a month at the time of investigation, then the individuals were placed in the “Participating” group for that social activity. In order to investigate the association between higher participation and EQ-5D, we added up the number of social activity participated in by each individual to form “Social activity count” variable and evaluate the association with health-related quality of life. In addition, we also made “Social activity type” variable to compare the amount of association between the four kinds of social activities (religion, relationship, leisure, and charity).

### Statistical analysis

One-way analysis of variance (ANOVA) was used to analyze statistical differences in EQ-5D results between groups. The CHS 2011 was community represented data; therefore, we used a generalized linear mixed model to obtain more accurate results of the factors influencing health-related quality of life revealed by the CHS 2011 and, in particular, to analyze the associations among the level of participation in social activities, the type of social activity, and the EQ-5D assessment by considering characteristic of area where the survey was performed. We used an unstructured model for the covariance structure, which calculated each correlation separately. All statistical analyses were performed using SAS 9.3 (SAS Institute, Inc., Cary, NC).

## Results

Table 1 presents the study participants' socio-demographic characteristics. The total number of participants was 209,315. In the general population, the group who participated in one social activity ranks first, followed by the group who participated in two social activities (one = 37.4 %, two = 26.0 %). Male groups had the same order of ranking (one = 37.1 %, two = 27.5 %). However, the female group that had participated in none of the social activities ranked second (one = 37.6 %, zero = 26.6 %).

**Table 1:** Socio-demographic characteristics Unit: N (weighted %)

	Total		Male		Female	
Social activity^b^ count						
4	4476	(2.4)	1917	(2.2)	2559	(2.7)
3	16,317	(8.7)	7623	(8.7)	8694	(8.7)
2	50,775	(26.0)	24,367	(27.5)	26,408	(24.5)
1	81,326	(37.4)	37,074	(37.1)	44,252	(37.6)
0	56,421	(25.6)	23,550	(24.5)	32,871	(26.6)
Age group						
19–34	39,904	(28.5)	18,150	(29.6)	21,754	(27.4)
35–49	60,892	(33.6)	28,882	(34.6)	32,010	(32.7)
50–64	57,766	(24.1)	26,501	(24.0)	31,265	(24.2)
≥ 65	50,753	(13.8)	20,998	(11.8)	29,755	(15.7)
Education						
Less than high school	82,660	(24.7)	29,378	(18.3)	53,282	(31.0)
High school graduate	71,365	(39.4)	35,980	(41.8)	35,385	(37.0)
College graduate	55,290	(36.0)	29,173	(40.0)	26,117	(32.0)
Perceived health status						
Good	164,909	(85.2)	78,690	(88.3)	86,219	(82.2)
Bad	44,406	(14.8)	15,841	(11.7)	28,565	(17.8)
Stress awareness						
Yes	55,365	(28.3)	24,374	(28.7)	30,991	(28.0)
No	153,950	(71.7)	70,157	(71.3)	83,793	(72.0)
Marital status						
Single	29,985	(21.8)	16,582	(25.9)	13,403	(17.7)
Separated/Divorced/Widowed	34,425	(12.2)	7529	(6.1)	26,896	(18.1)
Married	144,905	(66.0)	70,420	(68.0)	74,485	(64.1)
Family income (thousands won)						
< 1200	41,738	(10.9)	15,778	(8.9)	25,960	(12.8)
1200–3000	61,945	(27.2)	28,796	(27.1)	33,149	(27.3)
3000–4800	49,936	(27.2)	23,850	(28.4)	26,086	(25.9)
≥ 4800	55,696	(34.8)	26,107	(35.6)	29,589	(34.0)
Economic activity						
Yes	131,665	(63.6)	73,723	(78.2)	57,942	(49.1)
No	77,650	(36.4)	20,808	(21.8)	56,842	(50.9)
Depression diagnosis						
Yes	5310	(2.2)	1113	(1.1)	4197	(3.4)
No	204,005	(97.8)	93,418	(98.9)	110,587	(96.6)
Co-morbidity^a^ count						
≥ 3	16,934	(5.2)	4037	(2.8)	12,897	(7.5)
2	23,336	(8.0)	8697	(6.6)	14,639	(9.3)
1	46,701	(19.8)	22,222	(20.4)	24,479	(19.1)
0	122,344	(67.1)	59,575	(70.2)	62,769	(64.1)
Cultural facility^c^	5.15 ± 2.88		5.17 ± 2.91		5.13 ± 2.86	
Welfare facility^c^	12.61 ± 4.52		12.65 ± 4.52		12.58 ± 4.52	
Physical facility^c^	112.84 ± 9.57		112.93 ± 9.63		112.77 ± 9.51	
GRDP^c^	27.64 ± 8.25		27.64 ± 8.33		27.64 ± 8.19	
Hospital^c^	7590.47 ± 6705.49		7561.52 ± 6691.73		7614.32 ± 6716.74	
Total	209,315	(100.0)	94,531	(100.0)	114,784	(100.0)

^a^Co-morbidity included chronic and acute diseases, accidents or addictions, hypertension diagnosis, diabetes mellitus diagnosis, cerebral infarction diagnosis, cardiac infarction, angina pectoris diagnosis, arthritis diagnosis, osteoporosis diagnosis, tuberculosis diagnosis or asthma diagnosis

^b^Social activity included religion, relation, leisure, or charity

^c^Mean ± SD, GRDP: gross regional domestic product

Table 2 presents the average quality of life according to individuals’ level of participation in social activities. In the general population, we determined a strong positive correlation between the number of social activities an individual participated in and the average quality of life (zero = 89.30, one = 93.28, two = 95.25, three = 96.27, four = 96.85). Males and females showed similar results.

**Table 2:** Average quality of life according to amount of participation in social activities

	Total			Male			Female		
	Mean	Std. dev.	*p*-value	Mean	Std. dev.	*p*-value	Mean	Std. dev.	*p*-value
Social activity^b^ count									
4	96.85	7.52	<.0001	98.09	5.59	<.0001	95.93	8.57	<.0001
3	96.27	7.77		97.55	6.36		95.15	8.66	
2	95.25	9.35		97.09	7.42		93.56	10.55	
1	93.28	12.08		95.30	10.47		91.59	13.05	
0	89.30	17.77		91.25	17.39		87.90	17.91	
Age group									
19–34	98.05	5.48	<.0001	98.52	5.23	<.0001	97.65	5.66	<.0001
35–49	97.13	7.18		97.69	7.08		96.62	7.23	
50–64	93.76	11.34		95.57	10.48		92.22	11.80	
≥ 65	83.19	18.78		87.48	18.16		80.17	18.63	
Education									
Less than high school	86.95	16.98	<.0001	89.97	16.50	<.0001	85.29	17.02	<.0001
High school graduate	96.41	8.77		96.70	9.24		96.12	8.26	
College graduate	97.62	6.41		97.93	6.51		97.26	6.27	
Perceived health status									
Good	96.55	7.38	<.0001	97.54	6.41	<.0001	95.64	8.06	<.0001
Bad	79.81	20.05		82.33	21.20		78.41	19.24	
Stress awareness									
Yes	89.60	16.75	<.0001	92.64	15.52	<.0001	87.20	17.29	<.0001
No	94.22	11.46		95.80	10.24		92.89	12.24	
Marital status									
Single	97.39	7.94	<.0001	97.54	8.19	<.0001	97.20	7.62	<.0001
Separated/Divorced/Widowed	85.43	17.63		91.38	15.19		83.76	17.90	
Married	93.88	12.15		94.77	12.12		93.04	12.12	
Family income (thousands won)									
< 1200	83.87	18.53	<.0001	86.81	19.01	<.0001	82.09	18.00	<.0001
1200–3000	93.19	12.60		94.86	11.42		91.74	13.37	
3000–4800	96.13	9.02		97.38	7.68		94.98	9.95	
≥ 4800	96.81	8.09		97.89	6.58		95.85	9.12	
Economic activity									
Yes	95.84	8.40	<.0001	96.99	7.21	<.0001	94.38	9.50	<.0001
No	88.17	17.75		87.90	19.89		88.27	16.90	
Depression diagnosis									
Yes	79.80	20.74	<.0001	79.02	23.84	<.0001	80.00	19.83	<.0001
No	93.34	12.80		95.18	11.56		91.78	13.56	
Co-morbidity^a^ count									
≥ 3	74.98	21.07	<.0001	77.12	23.75	<.0001	74.32	20.11	<.0001
2	85.06	16.93		87.49	18.11		83.62	16.02	
1	92.07	12.67		93.58	12.34		90.69	12.80	
0	97.36	6.78		97.82	6.60		96.91	6.91	
Total	92.99	13.23	<.0001	94.99	11.91	<.0001	91.35	14.02	<.0001

^a^Co-morbidity included chronic and acute diseases, accidents, or addictions, hypertension diagnosis, diabetes mellitus diagnosis, cerebral infarction diagnosis, cardiac infarction, angina pectoris diagnosis, arthritis diagnosis, osteoporosis diagnosis, tuberculosis diagnosis or asthma diagnosis

^b^Social activity included religion, relationship, leisure, or charity

(adjusted by the number of cultural facility, welfare facility, physical facility, GRDP, Hospital in the region)

Table 3 presents the results of the generalized linear mixed model analysis, which assessed the association between amount of social activity participation and the quality of life. When all covariates were adjusted, in the general population those who participated in one or more social activities had higher EQ-5D scores compared with those who never participated in social activities. Furthermore, we found that more participation was positively associated with the higher EQ-5D (one = 1.939, two = 2.377, three = 2.439, four = 2.515, p for trend < 0.0001). Males and females followed similar trends as the general population.

**Table 3:** The association between amount of social activity and quality of life

	Total			Male			Female		
	β	S.E	*p*-value	β	S.E	*p*-value	β	S.E	*p*-value
Social activity^b^ count									
4	2.515	0.1607	<.0001	2.484	0.2322	<.0001	2.501	0.2210	<.0001
3	2.439	0.0936	<.0001	2.347	0.1313	<.0001	2.468	0.1321	<.0001
2	2.377	0.0645	<.0001	2.209	0.0918	<.0001	2.508	0.0903	<.0001
1	1.939	0.0567	<.0001	2.007	0.0819	<.0001	1.845	0.0781	<.0001
0	Ref.	-	-	Ref.	-	-	Ref.	-	-
P for trend			<.0001			<.0001			<.0001
Age group									
19–34	4.188	0.1114	<.0001	2.222	0.1532	<.0001	5.509	0.1636	<.0001
35–49	3.137	0.0892	<.0001	1.186	0.1211	<.0001	4.487	0.1349	<.0001
50–64	3.329	0.0721	<.0001	1.748	0.1024	<.0001	4.355	0.1031	<.0001
≥ 65	Ref.	-	-	Ref.	-	-	Ref.	-	-
P for trend			<.0001			<.0001			<.0001
Education									
Less then high school	−1.349	0.0795	<.0001	−1.506	0.1040	<.0001	−0.985	0.1221	<.0001
High school graduate	0.140	0.0598	0.0193	0.156	0.0789	0.0484	0.197	0.0900	0.0286
College graduate	Ref.	-	-	Ref.	-	-	Ref.	-	-
Perceived health status									
Good	8.467	0.0662	<.0001	8.566	0.0985	<.0001	8.258	0.0892	<.0001
Bad	Ref.	-	-	Ref.	-	-	Ref.	-	-
Stress awareness									
Yes	−3.187	0.0518	<.0001	−2.607	0.0734	<.0001	−3.623	0.0724	<.0001
No	Ref.	-	-	Ref.	-	-	Ref.	-	-
Marital status									
Single	−0.359	0.0840	<.0001	0.579	0.1150	<.0001	−0.375	0.1257	0.0029
Separation/Divorced/Widowed	−1.514	0.0664	<.0001	−0.143	0.1186	0.2276	−1.727	0.0848	<.0001
Married	Ref.	-	-	Ref.	-	-	Ref.	-	-
Family income (thousands won)									
< 1200	−2.029	0.0820	<.0001	−2.585	0.1194	<.0001	−1.567	0.1125	<.0001
1200–3000	−0.044	0.0633	0.4840	−0.185	0.0881	0.0361	−0.040	0.0899	0.6590
3000–4800	0.009	0.0633	0.8870	−0.051	0.0869	0.5588	−0.008	0.0906	0.9282
≥ 4800	Ref.	-	-	Ref.	-	-	Ref.	-	-
Economy activity									
Yes	2.640	0.0518	<.0001	4.246	0.0881	<.0001	1.960	0.0662	<.0001
No	Ref.	-	-	Ref.	-	-	Ref.	-	-
Depression diagnosis									
Yes	−5.428	0.1438	<.0001	−8.173	0.2925	<.0001	−4.674	0.1691	<.0001
No	Ref.	-	-	Ref.	-	-	Ref.	-	-
Co-morbidity^a^ count									
≥ 3	−10.344	0.1013	<.0001	−10.105	0.1734	<.0001	−10.362	0.1313	<.0001
2	−4.420	0.0838	<.0001	−3.805	0.1218	<.0001	−4.745	0.1161	<.0001
1	−1.587	0.0602	<.0001	−1.259	0.0809	<.0001	−1.893	0.0884	<.0001
0	Ref.	-	-	Ref.	-	-	Ref.	-	-

^a^ Co-morbidity included chronic and acute diseases, accident or addiction opportunity, hypertension diagnosis, diabetes mellitus diagnosis, cerebral infarction diagnosis, cardiac infarction, angina pectoris diagnosis, arthritis diagnosis, osteoporosis diagnosis, tuberculosis diagnosis or asthma diagnosis

^b^ Social activity included religion, relation, leisure or charity

(adjusted by the number of cultural facility, welfare facility, physical facility, GRDP, Hospital in the region)

**Fig. 1:**
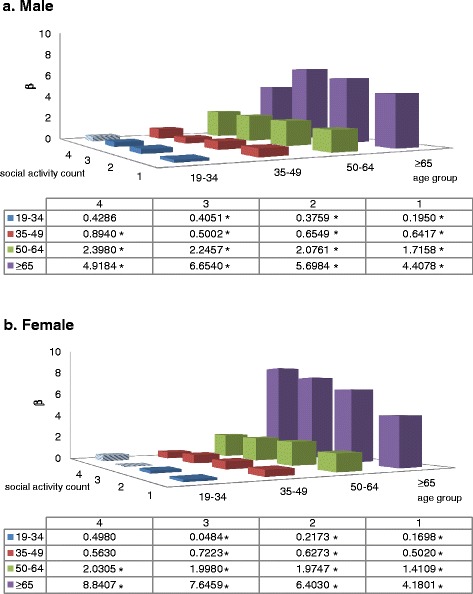
EQ-5D by social activity count by age group. **a** Male. **b** Female. Adjusted by education, perceived health status, stress awareness, marital status, family income, economic activity, depression diagnosis, co-morbidity, cultural facility, welfare facility, physical facility, GRDP, Hospital

Figure 1 shows the association between amount of social activity participation and quality of life by gender and age-group. In both male and female groups, when individuals participated in one social activity, social activity was more strongly associated with EQ-5D in elderly individuals (males: 19–34 years = 0.195, 35–49 years = 0.642, 50–64 years = 1.716, ≥65 years = 4.408; females: 19–34 years = 0.170, 35–49 years = 0.502, 50–64 years = 1.411, ≥65 years = 4.180). Of note, females had a more rapid increase in EQ-5D going from the younger age groups to the over 65 age group; in contrast, the male group had more of a gradual increase between age groups.

**Fig. 2:**
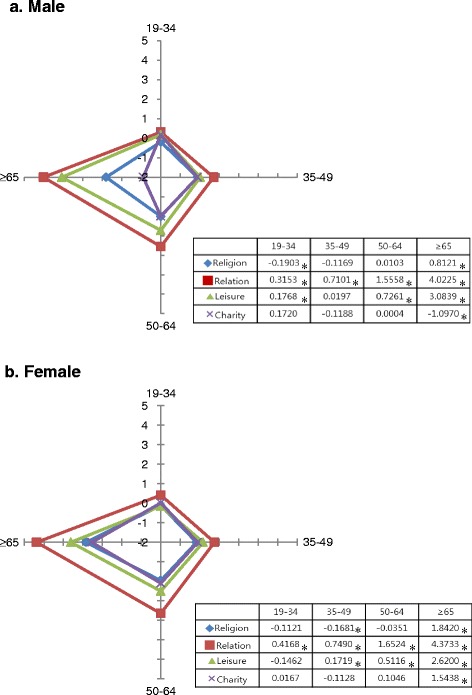
EQ-5D by social activity type by age group. **a** Male. **b** Female. Adjusted by education, perceived health status, stress awareness, marital status, family income, economic activity, depression diagnosis, co-morbidity, cultural facility, welfare facility, physical facility, GRDP, Hospital

Figure 2 shows the association between each type of social activity and quality of life according to gender and age group. In males, those aged 65 or older who participated in relationship organizations had the highest EQ-5D, followed by leisure activities, and religious activities (Relationship = 4.023, Leisure = 3.084, Religion = 0.812). The other age and gender groups had similar results. Specifically, we found that females who participated in relationship organizations had a much higher EQ-5D scores than those who participated in other social activities (Females ≥65 age groups; Relationship = 4.373, Leisure = 2.620, Religion = 1.842, Charity = 1.544).

## Discussion

The aim of this study was to assess whether participation in social activities was associated with health-related quality of life according to gender and age group [13, 14]. Our main findings indicated that participation in social activities was positively associated with health-related quality of life in all groups regardless of age or gender. Subsequently, we focused on the amount of participation and the types of social activities (religion, relationship, leisure, charity) most associated with high health-related quality of life.

Those who participated in social activities had higher health-related quality of life than those who did not, as supported by previous studies [10]. We also found that people who participated in more social activities had higher health-related quality of life than those who participated in less or none at all.

Our results suggested that the biggest differences were seen in the elderly between those who participated in social activities and those who did not, irrespective of gender. This finding supports previous studies, which indicated that for the elderly, participation in social activities was more important for increasing the quality of life than in the younger population [15] and this trend was more pronounced in elderly females. In the male group, the difference in EQ-5D between those who participated in social activities versus those who did not increased gradually by age group. On the other hand, females under 65 years of age had small differences in EQ-5D between those who participated in social activities and those who did not by age group, but after 65 years of age, there was a sharp increase in EQ-5D between those who participated and those who did not. In addition, females ≥65 years of age had a larger EQ-5D increase than males and this difference between genders was maintained when considering the number of social activities participants were involved in. Therefore, we conclude that for females, participation in social activities was more specifically associated with higher quality of life [15].

When we focused on the type of social activities, the social activity most associated with quality of life was relationship and the second was leisure activity [16]. This finding supports a previous study that indicated that the most effective social activity for increasing health-related quality of life is relationship-based [17]. The third most effective social activity was religious activity. In contrast to the present study, results from other studies did not show a statistical association [18, 19]. Furthermore, in the 35 to 49 age group, females reported a negative effect on health-related quality of life. One explanation for the lack of consistency among studies is the difference in the level of religious depth. Further, there is religious diversity in Korea, but the survey did not include the different kinds of religions/practices.

A previous study found no statistically significant association among all gender and age groups with respect to participation in charity activities [20]. In contrast, we noted a positive correlation between females in the ≥65 year age group who participated in charity activities versus those who did not. We speculate that the difference in these findings is due to the fact that the definition of charity activity was not obvious and the number of participants was too small in the Korean population.

When we stratified the population by gender and age group, there were differences in the effective type of social activities between each cluster. For men in the 19 to 34 and the 50 to 64 age groups, relationship was the most effective and leisure was the second most effective social activity for health-related quality of life. For men in the 35 to 49 age group, only relationship was effective. For men in the ≥ 65 age group, all social activities except charity were effective for improving quality of life. For women in the 19 to 49 age group, relationship was the only effective social activity. For women in the 50 to 64 age group, relationship and leisure were both positively associated with quality of life. In the ≥65 age group, all kinds of social activity had a positive effect on QOL. Therefore, we recognize that the elderly population derives more benefit from all forms of social activity compared to younger individuals [15]; for women, a greater variety in social activities was most effective.

To our knowledge, there are few studies that have recognized the association between social activities and health-related quality of life in the general population (especially among different types of social activities) [21], although several studies demonstrated an association between social activities and life satisfaction [22]. Our study demonstrated that participating in social activities is associated with better quality of life, which included participants’ health status [23]. Furthermore, we investigated whether participation in more social activities increased the health-related quality of life. Specifically, we demonstrated the association between the type of social activity and health-related quality of life according to gender and age group.

Our study had some limitations. First, we performed a cross-sectional study; therefore, we could not determine any mutual effect between social activity and health-related quality of life. Second, the survey did not distinguish among religious practices, so we could not determine differences in effectiveness, if any, between religious practices. Third, the survey did not investigate the depth of religious activity, so that the results of this study could not be linked with previous studies. Fourth, the survey did not define “charity activity”, so participants might not have classified their activities appropriately.

In conclusion, despite some limitations, our findings have novel implications. Currently, people desire not only economic wealth, but good health as well. In keeping with this trend, the government encourages participation in social activities. However, the emphasis has been mainly on the elderly population, and has focused on volunteer work, leisure, and cultural programs in senior centers. Interestingly, the most effective type of social activity was friendship-related, which was effective for all age groups, and especially for females [15]. Importantly, the government has not considered the under 65 age groups. Our study indicates that the Korean government should consider expanding policies that encourage participation by all age groups in social activities, especially those that are friendship-related. Finally, it is important that individuals participate in various social activities, especially those that include relationships, to enhance the quality of life.

## Abbreviations

HRQOL: Health related quality of life
CHS: Community Health Survey
EQ-5D: EuroQol five dimensions questionnaire
EQ-VAS: EuroQol visual analogue scales
